# Measurement of muscle passive stiffness in vibration-exposed groundskeepers

**DOI:** 10.1007/s00420-026-02214-6

**Published:** 2026-04-30

**Authors:** Nathan Chen, Seunghyeon Yang, Justin Leach, Jonghwa Oh

**Affiliations:** 1 Department of Environmental Health Sciences, School of Public Health, University of Michigan, Ann Arbor, MI USA; 2 Department of Environmental Health Sciences, School of Public Health, University of Alabama at Birmingham, Birmingham, AL USA; 3 Department of Biostatistics, School of Public Health, University of Alabama at Birmingham, Birmingham, AL USA

**Keywords:** Hand-arm vibration, Hand-arm vibration syndrome (HAVS), Groundskeepers, Myotonometer, Passive stiffness

## Abstract

**Objective:**

Currently, there is no standardized method to quantify musculoskeletal health of hand-arm vibration syndrome (HAVS). This study evaluated passive stiffness (PS) of the abductor pollicis brevis (APB) muscle among groundskeepers and examined the potential relationship of the PS with lifetime hand-arm vibration (HAV) exposure of the workers.

**Methods:**

PS of APB muscle was measured using a myotonometer among 17 groundskeepers and 10 office workers for 3 days. Study participants’ HAV exposure, demographic information, social history, and health-related information were pulled from our previous studies conducted in parallel with the present study. Linear mixed models were employed to estimate the association between lifetime HAV exposure dose and PS.

**Results:**

The average PS for right and left hands was 509.7 N/m (range 402.2–752.9 N/m) and 501.7 N/m (range 380.4–660.7 N/m), respectively, in the exposure group and 422.0 N/m (range 365.0–517.2 N/m) and 430.3 N/m (range 359.2–591.9 N/m), respectively, in the reference group. A significant positive association between lifetime HAV exposure and PS was observed in the multivariable linear mixed model after adjusting for age, body mass index, and race/ethnicity (right hand: β = 6.972 and p-value = 0.0128; left hand: β = 9.039 and p-value = 0.0108).

**Conclusions:**

A significant association between lifetime HAV exposure and PS was found, supporting PS measurement using myotonometry as a promising health indicator for muscular disorders induced by HAV.

**Supplementary Information:**

The online version contains supplementary material available at 10.1007/s00420-026-02214-6.

## Introduction

Excessive exposure to hand-arm vibration (HAV) is associated with the development of the hand-arm vibration syndrome (HAVS), a complex of vascular, neurological, and musculoskeletal disorders (Bovenzi 1998; Bovenzi et al. 1994; Heaver et al. 2011; Pelmear et al. 1993). The vascular and neurological symptoms may include blanching, coldness, numbness, tingling, and loss of sensation on upper extremities (House and Thompson 2015; Ye and Griffin 2018). HAV exposure may also affect the bones, joints, muscles, and tendons, inducing musculoskeletal disorders (MSDs) and symptoms such as loss of finger dexterity and decrease in grip strength (Mahbub et al. 2015). HAVS can become irreversible and lead to disability of upper extremities if preventive measures are not taken in early stages. (Bodley et al. 2015; House et al. 2009; Zimmerman et al. 2020). It is estimated that approximately 2.5 million workers are exposed to HAV from operating power tools, with a 20–50% prevalence of HAVS in the US (Palmer et al. 2001; USNSCASPO 2022). Industries in which workers are potentially exposed to HAV include forestry, construction, groundskeeping, agriculture, and dentistry (Banga et al. 2021; Chowdhry and Sethi 2017; Palmer et al. 2001).

Occupational Safety and Health Administration (OSHA) regulations on vibration exposure do not exist in the US. Standards and guidelines related to HAV are available, including American National Standard Institute (ANSI) S2.70–2006 and American Conference of Governmental Industrial Hygienists (ACGIH) threshold limit values (TLVs), and EU Directive 2002/44/EC (American Conference of Governmental Industrial Hygienists 2018; American National Standards Institute 2006). The ANSI standard, ACGIH guideline, and the EU Directive all set the daily exposure limit value at 5 m/s^2^ and the daily exposure action value at 2.5 m/s^2^ for an 8-hour work shift (American Conference of Governmental Industrial Hygienists 2018; American National Standards Institute 2006; European Parliament and the Council of the European Union 2002). The exposure limit values were established based on epidemiologic studies which investigated the association between vascular disorders of HAVS (i.e., vibration-induced white finger, VWF) and HAV exposure (Bovenzi 1998). There is a critical need for the establishment of a dose-response relationship between HAV exposure and muscular component of HAVS, which requires a reliable health indicator for the muscular health (Mahbub et al. 2015).

A reliable health indicator for the muscular health of HAVS should be able to quantify the symptoms among HAVS patients or the early health changes among HAV-exposed individuals. One of the main reasons for the impairments in dexterity and decrease in grip strength among HAVS patients is the muscle stiffness caused by HAV-induced muscle fibrosis (Kostyshyn et al. 2019). The muscle fibrosis has been shown to respond to injuries within 2–3 weeks, indicating that measuring the muscle stiffness could be a sensitive measure to detect early symptoms of muscular disorders (Mahdy 2019). A human biopsy study further showed that the abductor pollicis brevis (APB) muscle is highly sensitive and specific to HAV-induced injuries (Necking et al. 2004). In recent years, myotonometry has been validated as a non-invasive technology to quantify passive stiffness (PS) on the surface layer muscles (Feng et al. 2018; Ghatas et al. 2021; Hobson-Webb 2020; Janczyk et al. 2020; Kelly et al. 2018; Klich et al. 2019, 2020; Koga et al. 2021; Lee et al. 2021; Maeda et al. 2021; Taş et al. 2021), including APB muscles to evaluate muscular health (Pille et al. 2015, 2016). Although the current literature strongly supports that PS measurement of the APB muscle using myotonometry can be a promising indicator to help establish the dose-response relationship between HAV exposure and muscular disorders of HAVS, relevant studies remain limited.

Workers in the grounds maintenance industry operate multiple power tools and equipment, such as grass trimmers, blowers, chainsaws, and mowers on a regular basis (Chen et al. 2025b; Oh et al. 2023; Palmer et al. 2001). While groundskeepers are at risk of HAVS, the symptomatic characteristics of HAVS by geographical differences are little known. It is estimated that there are more than 60,000 groundskeepers in the southeastern US (i.e., Alabama, Georgia, South Carolina, Mississippi, and Louisiana) with an expected growth rate of 9.7% from 2022 to 2032 (Bureau of Labor Statistics 2024; Projections Central 2024). Recent studies conducted in warm environments (i.e., a subtropical or a tropical climate) found a higher prevalence of musculoskeletal symptoms than vascular and neurological symptoms among groundskeepers (Su et al. 2012; Xiao et al. 2019; Yang et al. 2023), showing the need to further investigate the relationship between HAV exposure and musculoskeletal health effects in warm environments.

In our previous studies, we evaluated lifetime HAV exposure dose of groundskeepers in the Southeastern US (Chen et al. 2025b) and the relationship between the lifetime exposure and finger blood flow as a vascular health indicator of HAVS (Chen et al. 2025a). The present study aimed to evaluate APB muscle stiffness among the groundskeepers and examine the potential relationship of the passive muscle stiffness with lifetime HAV exposure reported in our previous study (Chen et al. 2025b). The study hypothesized that a positive relationship exists between HAV exposure and the PS of APB muscle among the HAV-exposed workers.

## Methods

### Study population and monitoring plan

IRB approval was obtained prior to the study (UAB IRB #300008388). Groundskeepers and office workers over the age of 18 from two universities in Alabama state were recruited as the exposure group and the reference group, respectively. This study was conducted in parallel with our HAV exposure assessment study and details on HAV measurement was previously published (Chen et al. 2025b). A total of 6 days for PS measurement were scheduled on the same vibration monitoring days for each study participant: the first 3 days were scheduled within a week and approximately a month later, another 3 days were scheduled within a week to take monthly variations into account. The muscle stiffness measurement was scheduled prior to the start of a work shift for each study participant on the same day as the vibration measurement.

## Measurement instrument and questionnaires

**Fig. 1:**
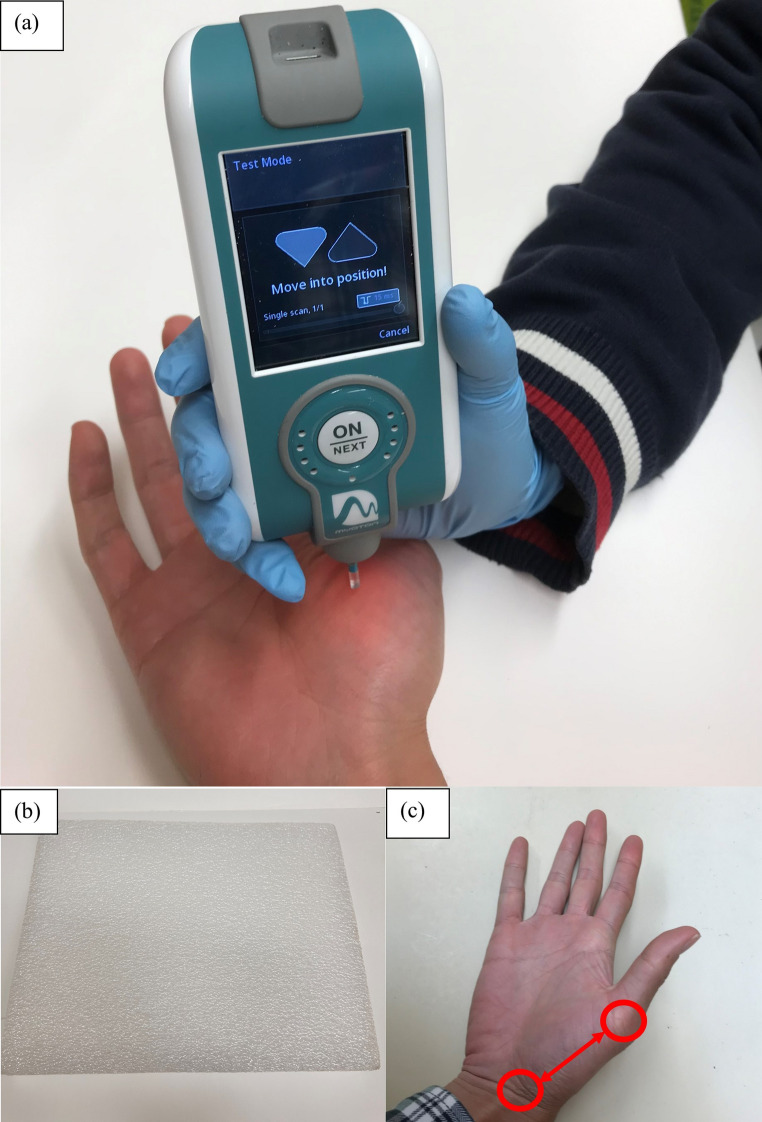
Passive stiffness (PS) of abductor pollicis brevis (APB) muscle was measured by **a** myotonometer on **b** an assigned surface at **c** the midpoint between thumb carpometacarpal joint and metacarpophalangeal joint

Myotonometer (MyotonPro, Myoton, Tallinn, Estonia) was used to measure PS of the APB muscle on the participant’s both hands. The myotonometer provides a 0.4 N impulse for 15 milliseconds to induce a damped natural oscillation of the tissue and PS is determined by formula 1 as follows:

1 $$\:PS\left(N/m\right)=\frac{a\times\:{m}_{\mathrm{probe}}}{\varDelta\:l}$$

where a is the maximum acceleration of the damped oscillation (m/s²), m_probe_ is the mass of the probe (kg), and Δl is the amplitude of the displacement at the end of the impulse time (m) (Klich et al. 2019). Prior to the measurement, the participants were asked to rest their hands/arms, forearms supinated, and hands supported an assigned surface on a desk in a seated position for minutes to avoid muscle activations (Davidson et al. 2017). The midpoint between thumb carpometacarpal joint and metacarpophalangeal joint was marked on both hands to ensure a consistent measurement location, and the probe of the myotonometer was placed perpendicular to the skin above the marked positions shown in Fig. 1. Three consecutive measurements were taken each day and averaged. The coefficient of variation of the myotonometer reported by the manufacturer is less than 3%. The triplicate measurements were verified to have the coefficient of variation below 3% to ensure the measurement reliability. In the current literature on the thenar muscle PS measurement, the reported standard measurement error was between 16.4 and 23.8 N/m (Davidson et al. 2017).

A questionnaire adapted from the EU Risks of Occupational Vibration Exposures (VIBRISK) project was used to obtain the study participants’ health status (Griffin and Bovenzi 2007). Questions on hand functional movement related to muscular health included difficulties in turning a doorknob or lever, opening a tight jar lid, putting on a jacket or pullover, fastening buttons, handling and picking up coins, and pouring from a jug or a pot. Questions regarding the participants’ demographic characteristics, social history, health-related information, and personal vibration exposure history were reported in our previous study (Chen et al. 2025a, b). For vibration exposure history, information on tool types used by each individual and corresponding operation duration was obtained. Lifetime cumulative HAV exposure dose (a_hv−lifetime_) was estimated by multiplying the exposure duration and the vibration magnitude in vibration total value (a_hv_) determined based on the real-time vibration measurement for each tool used by individual groundskeepers as reported in our previous study (Chen et al. 2025b).

### Statistical analysis

All the PS data was downloaded using MyotonPro version 5.0.0.257 (Myoton, Tallinn, Estonia). The descriptive statistics of participants’ health status and PS were analyzed in R Version 4.3.0. The data on demographic characteristics, social history, health-related information, and a_hv−lifetime_ was obtained from our previous studies for statistical analyses (Chen et al. 2025a, b). The first three-day average a_hv−lifetime_ and PS for each participant between the exposure and reference groups were compared for both hands using two-sample t test with unequal variance. The association between PS and a_hv−lifetime_ were investigated for both hands using a linear mixed model with study participant as random effects and the first three days of PS sampling conducted within a week as three repeated measures (nlme Version 3.1–162, R Version 4.3.0). The individual a_hv−lifetime_ values were divided by 10,000 to change the unit from hours·m/s^2^ to 10000·hours·m/s^2^ before model fitting for better interpretability of the regression coefficients. Three models, one without adjusting for any potential confounders, another with adjusting for demographic characteristics (i.e., race/ethnicity and age), and the other with adjusting for both demographic characteristics and social/health-related history, were tested. For the third model, analyses were conducted for potential confounders related to social history and health-related information (i.e., hand surgery, alcohol, smoking, diabetes, hypertension, and body mass index (BMI)), through fitting the model while adjusting for demographic characteristics and each of the social history and health-related variables (Collins et al. 2018; Degens et al. 2015; Filgueiras et al. 2023; Gueugneau et al. 2016; Hernández et al. 1999; Urbano-Márquez and Fernández‐Solà 2004). Only the significant health status variable(s) were considered in fitting the third model to avoid overfitting. Q-Q plots and the Shapiro-Wilk normality test were also conducted for each model to examine including the fitness of the data to the models. Additional analyses with log-transformed PS were conducted to evaluate the effect of the abnormality on the model when an obvious deviation from the diagonal line in the Q-Q plots was observed or the Shapiro-Wilk normality test was significant in the model. The study further explored the exposure-response relationship between PS and a_hv−lifetime_ through the internal comparison analysis within the exposure group only when the coefficients of a_hv−lifetime_ in the original models were observed to be significant. Two additional linear mixed model tests were conducted to evaluate the robustness of the original models above using the same settings, including the first analysis using PS for all six days as the dependent variable instead of using PS for the first three days and the second analysis with an interaction term between a_hv−lifetime_ and BMI added to the original model adjusted for both demographic characteristics and social/health-related history. The significance level of the two-sample t test and the coefficient in the linear mixed models were set at *p* < 0.05.

## Results

### Demographic characteristics, social history, health-related information, and lifetime HAV exposure dose

17 groundskeepers and 10 office workers were recruited in the study. As reported in our previous study, three to six days of exposure data was obtained from the participants, except for two participants: one participant for two days and the other participant for one day (Chen et al. 2025b).

The range of vibration total value (right hand a_hv_/ left hand a_hv_) of eleven power tools used by the seventeen groundskeepers was 4.2–6.3/3.8-7.0, 3.0-5.5/3.1–5.1, 1.0-2.8/1.7–3.3, 5.2–8.3/4.4–7.3, 1.9–2.7/0.8–2.3, 5.0-7.8/5.0-7.8, 4.6–5.8/3.7–3.8, 2.5–6.5/2.5–3.6, 2.2–3.1/2.1–3.1, 2.0–2.0/1.7–1.7, 8.0–8.0/3.4–3.4 m/s^2^ for grass trimmers, riding mowers, seating mowers, edgers, backpack blowers, push mowers, pole saws, hedgers, riding blowers, and chainsaws, respectively, as reported in our previous study (Chen et al. 2025b).

Demographic characteristics, social history, heath-related information, and HAV data (a_hv−lifetime_) previously reported are shown in Table 1 (Chen et al. 2025a, b). The average a_hv−lifetime_ was 76520.6 and 61955.5 h·m/s^2^ among the exposure group and 2306.2 and 2205.8 h·m/s^2^ among the reference group for the right and left hands, respectively, with significant differences between groups observed for both hands (p-value < 0.01). Four study participants from the reference group reported that they used grass trimmers, push mowers, or handheld blowers to maintain the yards at their house. One participant in the reference group reported occasional use of a chainsaw years ago when he was a firefighter.

**Table 1:** Demographic characteristics, social history, health-related information, lifetime hand-arm vibration exposure dose (a_hv−lifetime_), and muscle passive stiffness (PS) in the exposure group and the reference group

Number of participants^a^ (African American/ Caucasian/Hispanic)	Exposure group	Reference group	
	17 (4/7/6)	10 (2/5/3)	
Biological sex (Male/Female)^a^	17/0	10/0	
Dominant hand (Right/Left)^a^	15/2	10/0	
Long-term Medication or hand surgery ^b^ (Hypertension/Diabetes/hand surgery)	5 (3/1/2)	2 (1/0/1)	
Smoke/Alcohol use ^b^ (Smoke/Alcohol)	12 (3/12)	8 (6/7)	
Age in years (Range) ^a^	44 (23 ~ 67)	43 (22 ~ 73)	
Body Mass Index, BMI (Range)^b^	30 (22 ~ 42)	29 (21 ~ 36)	
Years of working in industries where involved the regular use of vibrating equipment (Range)^a^	13 (1 ~ 27)	0 (0 ~ 0)	
	Mean ± SD (Range)		*p*-value
Right hand			
_ℎ−lifetime_ in hours·m/s^2a*^	76520.6 ± 53224.5 (1459.5 ~ 154234.9)	2306.2 ± 4889.3 (0 ~ 15408.9)	< 0.001
PS in N/m^c*^	509.7 ± 82.3 (402.2 ~ 752.9)	422.0 ± 50.4 (365.0 ~ 517.2)	0.002
Left hand			
_ℎ−lifetime_ in hours·m/s^2a*^	61955.5 ± 43406.6 (1253.1 ~ 140702.6)	2205.8 ± 4847.1 (0 ~ 15217.2)	< 0.001
PS in N/m^c*^	501.7 ± 78.4 (380.4 ~ 660.7)	430.3 ± 65.8 (359.2 ~ 591.9)	0.019

^a^Data reported in our previous study (Chen et al. 2025b)

^b^Data reported in our previous study (Chen et al. 2025a)

^c^Showing mean of the first three-day sampling by each participant

*p-value < 0.05 under the two-tail t-test with unequal variance

### The thenar muscle PS

All participants completed six days of PS measurement. Table 1 shows the descriptive statistics for participants’ PS measurement in the exposure group and the reference group. The first three-day average PS among the exposure group was 509.7 N/m (range 402.2–752.9 N/m) and 501.7 N/m (range 380.4–660.7 N/m) for the right and left hands, respectively. For the reference group, the first three-day average PS was 422.0 N/m (range 365.0–517.2 N/m) and 430.3 N/m (range 359.2–591.9 N/m) for the right and left hands, respectively. There were significant differences of 87.7 and 71.4 N/m for the right (p-value = 0.002) and left hands (p-value = 0.019), respectively, in PS between the exposure group and reference group. The individual PS data for all 27 study participants for the entire measurement period is available in Supplementary Table S1. The six-day average PS was 489.4 N/m (range 402.1–706.1 N/m) for the right hand and 485.8 N/m (range 376.5–613.0 N/m) for the left hand among the exposure group. For the reference group, the six-day average PS was 415.4 N/m (range 533.6–521.7 N/m) for the right hand and 421.2 N/m (range 357.6–559.1 N/m) for the left hand.

## Hand functional movement difficulties in relation to muscular health

Among the exposure group, 2 people reported experiences of difficulties in turning a doorknob or lever, 5 people in opening a tight jar lid, 1 person in putting on a jacket or pullover, 4 people in fastening buttons, 2 people in handling and picking up coins, and 2 people in pouring from a jug or a pot. One person who had a hand surgery from the reference group reported difficulties in opening a tight jar lid, fastening buttons, and handling and picking up coins. Details on the survey results can be found in Table S2.

## Association between lifetime HAV exposure (a_hv−lifetime_) and passive stiffness (PS)

The association between a_hv−lifetime_ and PS is shown in Table 2. There was a significant association between lifetime HAV exposure and PS in all three models. The first model, without adjustment, showed a coefficient (β) of 7.850 for the right hand (p-value = 0.0050) and 8.476 for the left hand (p-value = 0.0127). The second model, adjusted for demographic characteristics, yielded a β of 7.940 (p-value = 0.0087) for the right hand and 9.288 (p-value = 0.0092) for the left hand. The last model, adjusted for demographic characteristics and social/health-related history, had a β of 6.972 (*p*-value = 0.0128) for the right hand and 9.039 (p-value = 0.0108) for the left hand. The interpretation of the β estimates from the above three models is that for every 10000 h·m/s^2^ increase in a_hv−lifetime_, we estimate a significant increase in PS by 7–8 N/m for the right hand and 8–9 N/m for the left hand. A significantly lower PS on the left hand was observed among African Americans than Caucasians in the model adjusted for both demographic characteristics and social/health-related history. Higher BMI was significantly associated with higher PS on the right hand in the model adjusted for both demographic characteristics and social/health-related history. Some deviations from the diagonal line in the Q-Q plots were observed, and the Shapiro-Wilk normality tests were significant throughout the models. Thus, additional analyses with log-transformed PS were conducted, as shown in Table S3. No obvious deviation from the diagonal line in the Q-Q plots were observed, and the Shapiro-Wilk normality tests were not significant throughout the models in the additional analyses. Because of the coefficients of a_hv−lifetime_ being significant, the internal comparison analysis with groundskeepers only was conducted. The results showed that no significant associations were observed in the coefficients of the a_hv−lifetime_ for both hands, as shown in Supplementary Table S4. The results of the two additional tests are shown in Supplementary Tables S5 and S6. In the first analysis with all six-day PS, significant associations between a_hv−lifetime_ and PS were observed in all three models (Table S5). In the second analysis with the interaction term between a_hv−lifetime_ and BMI added, statistical significances were observed in the association between lifetime HAV exposure and PS and the interaction between BMI and a_hv−lifetime_ in the left-hand model only (Table S6).

**Table 2:** Association between lifetime hand-arm vibration exposure dose (a_hv−lifetime_) and passive stiffness (PS) after adjusting for none, demographic characteristics, or demographic and health characteristics in the linear mixed model

		β, *p*-value (95% CI)	
	Simple^a^	Demographic^a, b, d^	Demographic +health status^a, c, d, e^
Right hand			
_ℎ−lifetime_	7.850, 0.0050** (2.601, 13.10)	7.940, 0.0087** (2.219, 13.66)	6.972, 0.0128* (1.643, 12.30)
Age		0.3226, 0.7855 (-2.106, 2.751)	0.4434, 0.6840 (-1.790, 2.6771)
Race/ethnicity-Afr		-27.36, 0.4819 (-106.7, 51.96)	-81.42, 0.0679 (-169.4, 6.531)
Race/ethnicity-His		-9.042, 0.8049 (-84.04, 65.96)	-21.47, 0.5294 (-91.30, 48.35)
BMI			7.497, 0.0330* (0.6671, 14.33)
AICc, BIC	909.1, 918.1	895.6, 910.4	889.0, 905.6
Left Hand			
_ℎ−lifetime_	8.476, 0.0127* (1.976, 14.98)	9.288, 0.0092* (2.547, 16.03)	9.039, 0.0108* (2.317, 15.76)
Age		0.0269, 0.9810 (-2.283, 2.337)	0.0724, 0.9484 (-2.227, 2.372)
Race/ethnicity-Afr		-66.27, 0.0787 (-140.8, 8.258)	-94.37, 0.0401* (-184.0, -4.702)
Race/ethnicity-His		-41.71, 0.2384 (-113.1, 29.66)	-48.78, 0.1743 (-120.9, 23.36)
BMI			3.833, 0.2594 (-3.044, 10.71)
AICc, BIC	921.1, 930.1	904.6, 919.4	901.5, 918.1

^a^First three-day sampling results used for the linear mixed model as repeated-measures data

^b^Adjusting for age and race/ethnicity

^c^Adjusting for age, race/ethnicity, and BMI

^d^Information of age and race/ethnicity were obtained from our previous study (Chen et al. 2025b)

^e^Information of BMI was obtained from our previous study (Chen et al. 2025a)

**p-value < 0.01

*p-value < 0.05

In alignment with statistical literature, we estimated power for a range of effects, including values significantly weaker and stronger than those observed, and which provides a reasonably comprehensive understanding of what effect sizes the study was powered to detect (Althouse 2021; Hoenig and Heisey 2001). Power analyses were based on the simple models in Table 2. Power estimates were based on a simulation study that included 10,000 replications for each scenario, i.e., each hand and each value of a_hv−lifetime_ coefficient, and we set the variance parameters to their estimated values from the respective model. For minimal a_hv−lifetime_ coefficients of 2.0-2.5, power was low at approximately 10% for both cases and both hands. In the range of observed a_hv−lifetime_ coefficient estimates of 7.85–8.50, power would be moderate at approximately 66%-74%. True effect parameter values of approximately 9.25 would be required to have 80% power given the sample size and assuming the observed variance parameter estimates. Full simulation study results are included in Supplemental Material Tables S7 and S8 and Figures S1 and S2. While useful in illustrating the power we would have to detect a range of effect sizes, power analysis does not provide information regarding the compatibility of the observed results with effect sizes of interest, e.g., 0 or no difference; interval estimates and hypothesis tests are better suited to this purpose (Greenland et al. 2016). Therefore, only confidence intervals and p-values were reported in Table 2 and further interpreted in the following discussion.

## Discussion

### Demographic characteristics, social history, health-related information, and lifetime HAV exposure dose

All study participants were male and study participants’ age distribution and the proportions of hand surgery and alcohol usage were comparable between groups as reported in our previous studies (Chen et al. 2025a, b). Studies showed that hypertension or diabetes may be associated with the morphological change on skeletal muscles (Filgueiras et al. 2023; Hernández et al. 1999). The higher prevalence of hypertension or diabetes in the exposure group reported in our previous study may thus affect PS in the same direction as muscular effects of HAV (Chen et al. 2025a). Smoking has also been associated with morphological change in skeletal muscles (Degens et al. 2015). The lower percentage of smoking in the exposure group reported in our previous study may be associated with lower PS, which may move to the opposite direction of the effects of HAV exposure (Chen et al. 2025a). A higher BMI in the exposure group as reported in the previous study may be linked to overweight or obesity and thus associated with skeletal muscle regeneration and fibrosis, which may affect PS in the same direction as the effects of HAV (Chen et al. 2025b; Collins et al. 2018). Therefore, long-term medication for hypertension or diabetes, smoking, and BMI were all listed as potential confounders. Only BMI among potential confounders related to social history and health-related information was significant in analyses and thus was controlled in fitting the final third model in Table 2 to avoid overfitting.

### The thenar muscle PS

A significant difference of PS was observed between the exposure group and the reference group, which supports our hypothesis that the higher levels of HAV exposure contribute to the higher PS values of APB muscle. In a Pille et al.’s study which measured PS on the APB muscle of garment workers and woodworking workers using myotonometry showed the average PS between 248.9 and 342.0 N/m with standard deviations between 48.3 and 72.6 N/m (Pille et al. 2015). Compared to the study, the groundskeepers in the present study showed higher average PS (501.7–509.7 N/m) with comparable standard deviations (78.4–82.3 N/m). One of the reasons may come from the difference in biological sex. Studies have shown that male’s leg and elbow muscle stiffness are generally higher than female’s (Granata et al. 2002; Lee and Ashton-Miller 2011; Morse 2011). The male s' thenar muscle stiffness may also generally be higher than the females', which may explain the lower average PS measured from both male and female participants in the Pille et al.’s study (Pille et al. 2015). In the present study, 13 groundskeepers’ right and left hands, 8 office workers’ right hand and 7 offices workers’ left hand showed a higher average of the first three days’ PS than their own average of all six days’ PS. We observed that during the first three days of PS measurement, the study participants seemed to be nervous and holding their hands tighter even though they were told to relax their hands during the measurement. Then, later during the rest three days of measurement sessions, they appeared to be more relaxed potentially because of their adaptation to the measurement protocols, which may have lowered the overall six-day average PS. Nonetheless, a significant difference of 74.0 N/m on the right hand and 64.6 N/m on the left hand in six-day average PS between the groups was observed (Table S1), showing that the pattern of positive association between HAV exposure and PS was consistent with the first three days' PS.

### Hand functional movement difficulties in relation to muscular health

In addition to the myotonometric method, the currently available quantitative tools which enable to assess the musculoskeletal health effects of HAVS include grip strength test, pinch strength test, and pegboard test (Cederlund et al. 1999; Mahbub et al. 2015). In the grip strength test and pinch strength test, the subject is asked to grip or pinch a dynamometer as hard as possible, which is a measure of the muscle strength of the hand (Poole and Mason 2007; Rudd et al. 2023). The Purdue pegboard test in which the test subject is asked to place pegs into the holes as many as possible in 30 seconds has been generally used in the evaluation of upper extremity motor function (Lawson 2019; Mahbub et al. 2015). While the Purdue pegboard test has shown the best performance in sensitivity and specificity among the three tests (Mahbub et al. 2015; Poole and Mason 2007), the specificity and the sensitivity of the Purdue pegboard test varied across studies (i.e., specificity: 44.8% to 85% and sensitivity: 78% to 95%) (Cederlund et al. 1999; Mahbub et al. 2015). Furthermore, those three tests are used to help diagnose HAVS, not to quantify early health changes induced by HAV. In the present study, one office worker reported hand functional difficulties which are related to muscular health, while 7 groundskeepers reported functional difficulties with their hands. Higher three-day average PS values were observed in the groundskeepers, regardless of whether they experienced difficulties with their hand function or not, indicating that PS may be an early, objective indicator of the muscular health of HAVS.

### Association between lifetime HAV exposure (a_hv−lifetime_) and passive stiffness (PS)

A significant association between a_hv−lifetime_ and PS was observed in all three models: the coefficient of a_hv−lifetime_ was from 6.972 to 7.940 for the right hand and from 8.476 to 9.288 for the left hand. If the average yearly HAV dose in the grounds maintenance industry is assumed to be 5886.2 h·m/s^2^ for the right hand (i.e., the average a_hv−lifetime_ of groundskeepers divided by average work year = 76520.6 h·m/s^2^ ÷ 13 years = 5886.2 h·m/s^2^ per year), then on average PS increases on the right hand from 4.1 to 4.7 N/m every year working as a groundskeeper. Similarly, on average PS increases from 3.8 to 4.3 N/m on the left hand every year working as this profession. Thus, it can be estimated that, for a groundskeeper who has been working for 25 years, an increase in PS between 95.0 and 117.5 N/m would be expected. This increase is 4.0 to 7.2 times the standard within-person measurement error (i.e., 16.4 and 23.8 N/m) reported by Davidson et al. (2017). The significantly lower PS on the left hand (i.e., p-value = 0.0401) and marginally lower PS on the right hand (i.e., p-value = 0.0679) were observed among the African American groundskeepers. The results may imply the potential difference in the baseline PS between different race/ethnicity groups, requiring more studies with larger sample sizes. In the analysis with log-transformed PS to evaluate the effect of the abnormality of the PS on the models, a significant association between a_hv−lifetime_ and PS was also observed in all three models with non-significant Shapiro-Wilk normality test. This supports the existence of the association between a_hv−lifetime_ and PS is not significantly affected by the abnormality of the PS. In the internal comparison analysis with the exposure group only, the coefficients of the a_hv−lifetime_ remained in the same direction but were smaller than the coefficients of thea_hv−lifetime_ in the original models (i.e., exposure and reference groups combined) for both hands, implying that the association still exists within the exposed group but with a smaller effect size. No significance in the coefficients of the a_hv−lifetime_ throughout the models may be due to both inadequacy of the sample size and the attenuated estimated effects compared to the primary analysis. In the first sensitivity test, the significant association between a_hv−lifetime_ and all six-day PS observed in all three models showed the consistent patterns with the relationship between the first three-day PS and a_hv−lifetime_. Considering the statistical significance in the coefficient of BMI and the corresponding improvement in the model fitting (i.e., a decrease in AICc value) especially in the right-hand model (Table 2), an additional analysis with the interaction variable between BMI and the lifetime HAV exposure was conducted to investigate the potential synergistic effect between BMI and the lifetime HAV exposure. While significances were observed in the coefficient of HAV lifetime exposure and that of the interaction variable in the left-hand model, no significance was observed in the right-hand model. Together with an inconsistency in model fitting (i.e., increased AICc and BIC values in the right-hand model and decreased AICc and BIC values in the left-hand model with the interaction variable added), the coefficient of the interaction between BMI and a_hv−lifetime_ should be carefully interpreted. The potential pathophysiological role of BMI in the prediction of PS in the dominant (right) hand and the possible interaction with HAV lifetime exposure need more investigations with a larger sample size in the future.

### Limitations and future research

There were limitations of the study. The frequency weighting system in ISO 5349-1 was employed in the present study. It is known that the hands and fingers are more sensitive to higher frequency vibration (Bovenzi 2012; Pitts et al. 2012), implying that the use of the frequency weighting system which weighs heavily on the lower frequency range (≥ 25 Hz) may underestimate the association between the PS and the a_hv−lifetime_. Second, more investigations with larger sample sizes and longer observation periods shall be followed to explore the relationship between a_hv−lifetime_ and PS among power-tool operators in the same occupation as well as other occupations. Third, investigations are also needed for female workers, considering the limited information about HAV-related exposure assessment and related muscular health effects for female workers.

## Conclusion

The present study (1) showed that the PS measurement on thumb muscle through the myotonomteric method may be a promising non-invasive, early muscular health indicator for HAVS among power tool operators, in particular groundskeepers, (2) will help better understand the pathophysiological mechanisms of the muscular disorders of HAVS that the vibration-induced injuries on the APB muscles may lead to muscle fibrosis (Lee and Ashton-Miller 2011; Mahdy 2019; Necking et al. 2004), and (3) showed the need of further investigations with larger sample sizes and prospective studies to help establish a reliable exposure-response relationship between HAV exposure and PS.

## Supplementary Information

Below is the link to the electronic supplementary material.

Supplementary Material 1

## Data Availability

Data are available on reasonable request. The data underlying this article cannot be shared publicly because of the privacy of individuals that participated in the study. Deidentified data can be shared on reasonable request to the corresponding author.
