# Supplementary material for: Measurement of muscle passive stiffness in vibration-exposed groundskeepers
Source: Int Arch Occup Environ Health. 2026 Apr 30;99(4):22. doi: 10.1007/s00420-026-02214-6 (PMC13132957; doi:10.1007/s00420-026-02214-6)
Supplement: Supplementary file 1 — Supplementary Material 1 [file 420_2026_2214_MOESM1_ESM.docx]

Table S1 Passive stiffness (PS) for the first three days and the second three days measured on the study participants’ right hand and left hand.

| PS/subjectID | PS for the first three days | | PS for all six days | |
| --- | --- | --- | --- | --- |
|  | Right hand | Left hand | Right hand | Left hand |
|  | Mean ±SD (Range) | | | |
| AA | 517.1±58.9  (461.3-578.7) | 425.6±64.4  (359.7-488.3) | 459.3±73.8  (394.3-578.7) | 415.4±42.5  (359.7-488.3) |
| AB | 552.7±32.8  (522.7-587.7) | 629.1±125.6  (531-770.7) | 525.3±38.4  (476.7-587.7) | 589.4±95.2  (507.7-770.7) |
| AC | 510.2±41.2  (465-545.7) | 492.9±60.1  (447.3-561.0) | 505.8±33.3  (465.0-545.7) | 485.3±39.4  (447.3-561.0) |
| AD | 510.4±49.6  (476.7-567.3) | 458.8±15.3  (448.7-476.3) | 521.4±56.3  (476.7-613.0) | 449.6±21.5  (412.0-476.3) |
| AE | 520.3±40.7  (475-553.7) | 531.3±54.0  (478-586) | 476.0±58.0  (401.7-553.7) | 494.7±53.1  (447.0-586.0) |
| AF | 443.7±18.4  (427-463.3) | 456.0±68.7  (390.7-527.7) | 402.1±48.1  (349.0-463.3) | 429.7±56.1  (366.0-527.7) |
| AG | 550.0±64.8  (475.3-591) | 660.7±83.0  (586.7-750.3) | 553.5±47.2  (475.3-591.0) | 613.0±77.6  (528.0-750.3) |
| AH | 413.2±38.2  (376.0-452.3) | 413.1±24.3  (392.0-439.7) | 403.4±27.7  (376.0-452.3) | 400.7±30.3  (358.0-439.7) |
| AI | 462.3±59.6  (413.3-528.7) | 420.6±77.1  (352.7-504.3) | 434.6±52.7  (382.7-528.7) | 388.9±60.6  (340.0-504.3) |
| AJ | 402.2±48.3  (348.3-441.7) | 380.4±14.2  (364.3-391.3) | 402.2±45.4  (348.3-463.3) | 376.5±35.5  (338.0-434.7) |
| AK | 580.0±68.9  (515.7-652.7) | 503.3±113.7  (379.0-602.0) | 564.8±46.9  (515.7-652.7) | 518.2±74.4  (379.0-602.0) |
| BA | 461±19.6  (439.3-477.7) | 513.7±46.2  (466.0-558.3) | 452.8±15.9  (439.3-477.7) | 515.3±35.4  (466.0-558.3) |
| BB | 752.9±112.5  (673.7-881.7) | 588.9±9.0  (580.3-598.3) | 706.1±97.0  (607.3-881.7) | 596.2±29.5  (564.7-651.3) |
| BC | 523.9±27.8  (492.3-544.7) | 511.3±23.9  (484.3-529.7) | 512.7±40.7  (449.0-558.0) | 519.6±19.7  (484.3-543.3) |
| BD | 507.2±51.3  (449.3-547) | 492.1±22.0  (469.3-513.3) | 485.9±43.3  (438.3-547.0) | 483.4±22.9  (460.3-513.3) |
| BE | 412.3±16.9  (397.3-430.7) | 462.2±27.8  (437.0-492.0) | 417.1±23.1  (390.0-452.7) | 418.5±54.5  (340.7-492.0) |
| BF | 544.7±89.0  (475.3-645.0) | 589.1±45.0  (544.0-634.0) | 497.1±77.0  (438.7-645.0) | 563.9±67.5  (448.0-634.0) |
| Average-exposure group | 509.7±82.3  (402.2-752.9) | 501.7±78.4  (380.4-660.7) | 489.4±76.3  (402.1-706.1) | 485.8±75.4  (376.5-613.0) |
| CA | 455.1±12.3  (447.3-469.3) | 453.2±42.2  (422.7-501.3) | 435.9±25.5  (395.7-469.3) | 442.7±29.7  (421.0-501.3) |
| CB | 383.9±50.3  (329.0-427.7) | 411.2±29.9  (392.7-445.7) | 367.8±45.7  (314.0-427.7) | 394.0±49.4  (309.3-445.7) |
| CC | 403.8±29.0  (376.7-434.3) | 381.4±59.9  (342-450.3) | 395.1±25.4  (369.0-434.3) | 384.4±52.8  (322.3-450.3) |
| CD | 517.2±82.3  (460.7-611.7) | 591.9±49.6  (541.0-640.0) | 521.7±84.7  (422.3-633.0) | 559.1±49.3  (507.3-640.0) |
| CE | 386.4±6.2  (381.3-393.3) | 359.2±19.8  (340.7-380.0) | 381.7±6.6  (375.3-393.3) | 364.5±24.4  (333.7-393.7) |
| CF | 390.8±10.5  (379.3-400.0) | 423.1±71.7  (355.7-498.3) | 417.4±32.7  (379.3-460.7) | 415.1±48.9  (355.7-498.3) |
| CG | 365.0±63.9  (299.0-426.7) | 421.2±15.8  (408-438.7) | 353.6±44.5  (299.0-426.7) | 400.7±24.7  (376.0-438.7) |
| CH | 384.4±33.6  (348.3-414.7) | 368.4±78.6  (308.0-457.3) | 383.9±27.0  (348.3-414.7) | 357.6±55.4  (308.0-457.3) |
| CI | 465.9±3.5  (462.3-469.3) | 451.0±10.3  (439.3-458.7) | 450.9±16.9  (431.7-469.3) | 464.3±26.1  (439.3-511.3) |
| CJ | 467.6±24.6  (450.3-495.7) | 441.8±15.0  (430.0-458.7) | 446.1±36.3  (400.7-495.7) | 429.6±28.2  (387.0-458.7) |
| Average-Reference group | 422.0±50.4  (365.0-517.2) | 430.3±65.8  (359.2-591.9) | 415.4±50.1  (353.6-521.7) | 421.2±58.9  (357.6-559.1) |

|  | Mean ±SD  (Range) | | | | | | | |  |
| --- | --- | --- | --- | --- | --- | --- | --- | --- | --- |
|  | Reference Group | Exposure group without hand functional difficulties | Exposure group with difficulties in turning a doorknob or lever | Exposure group with difficulties in opening a tight jar lid | Exposure group with difficulties in putting on a jacket or pullover | Exposure group with difficulties in fastening buttons | Exposure group with difficulties in handling and picking up coins | Exposure group with difficulties in pouring from a jug or a pot | |
| Right hand | N=10 | N=10 | N=2 | N=5 | N=1 | N=4 | N=2 | N=2 | |
| 𝑎_ℎ𝑣−lifetime_^a^ | 2306.2±4889.3 (0-15408.9) | 67747.1±49505.8  (1459.5-154234.9) | 129797.7±33928.0 (105807-153788.5) | 82081.5±63389.5 (10207-138469.8) | 59183.0±0  (59183.0-59183.0) | 78416.7±55914  (10207.0-138469.8) | 58007.0±67599.4 (10207.0-105807.0) | 138070.6± 564.6 (137671.4-138469.8) | |
| PS^b^ | 422.0±50.4 (365.0-517.2) | 497.5±60.4  (402.2-580.0) | 648.8±147.2  (544.7-752.9) | 540.2±126.4  (413.2-752.9) | 443.7±0  (443.7-443.7) | 556.0±135.4  (443.7-752.9) | 635.0±166.7  (517.1-752.9) | 508.8±2.3  (507.2-510.4) | |
|  |  |  |  |  |  |  |  |  | |
| Left hand | N=10 | N=10 | N=2 | N=5 | N=1 | N=4 | N=2 | N=2 | |
| 𝑎_ℎ𝑣−lifetime_^a^ | 2205.8±4847.1 (0-15217.2) | 55428.6±40535.1 (1253.1-117484.1) | 108614.4±45379.6 (76526.2-140702.6) | 60503.5±46588.3 (8699.8-118825.9) | 55738.0±0 (55738.0-55738.0) | 55623±33215.5 (8699.8-81528) | 42613.0±47960.5 (8699.8-76526.2) | 100177.0± 26373.6  (81528.0-118825.9) | |
| PS^b^ | 430.3±65.8 (359.2-591.9) | 510.6±84.8  (380.4-660.7) | 589.0±0.2  (588.9-589.1) | 475.7±70.4  (413.1-588.9) | 456.0±0  (456.0-456.0) | 482.3±72.6  (425.6-588.9) | 507.2±115.5  (425.6-588.9) | 475.4± 23.6  (458.8-492.1) | |

Table S2 Hand functional difficulties in relations to muscular health reported by both exposure and reference groups by hand side, matched by lifetime exposure dose (a_hv-lifetime_) and passive stiffness (PS).

^a^data reported in our previous study (Chen et al. 2025b)

^b^the mean of first three-day sampling by each participant

Table S3 Association between lifetime hand-arm vibration exposure dose (a_hv-lifetime_) and log-transformed passive stiffness (PS) after adjusting for none, demographic characteristics, or demographic and health characteristics in the linear mixed model.

|  |  | β, p-value  (95% CI) |  |
| --- | --- | --- | --- |
|  | Simple^a^ | Demographic^a, b, d^ | Demographic  +health status^a, c, d, e^ |
| Right Hand |  |  |  |
| 𝑎_ℎ𝑣−lifetime_ | 0.0161, 0.0032**  (0.0059, 0.0263) | 0.0164, 0.0052**  (0.0055, 0.0275) | 0.0148, 0.0079**  (0.0043, 0.0252) |
| Age |  | -0.0008, 0.7414  (-0.0039, 0.0054) | 0.0010, 0.6496  (-0.0034, 0.0053) |
| Race/ethnicity-Afr |  | -0.0562, 0.4541  (-0.2091, 0.0967) | -0.1530, 0.0791  (-0.3254, 0.0194) |
| Race/ethnicity-His |  | -0.0352, 0.6186  (-0.1798, 0.1094) | -0.0575, 0.3925  (-0.1943, 0.0794) |
| BMI |  |  | 0.0134, 0.0495*  (0.00003, 0.0268) |
| AICc, BIC | -76.05, -67.10 | -52.33, -37.55 | -45.73, -29.19 |
| Left Hand |  |  |  |
| 𝑎_ℎ𝑣−lifetime_ | 0.0180, 0.0092**  (0.0049, 0.031) | 0.0197, 0.0066**  (0.0061, 0.0333) | 0.0191, 0.0077**  (0.0056, 0.0326) |
| Age |  | -0.0001, 0.9526  (-0.0048, 0.0045) | -0.00004, 0.9870  (-0.0047, 0.0046) |
| Race/ethnicity-Afr |  | -0.1336, 0.0789  (-0.2841, 0.0168) | -0.1944, 0.0357*  (-0.3745, -0.0143) |
| Race/ethnicity-His |  | -0.0838, 0.2404  (-0.2278, 0.0602) | -0.0991, 0.1696  (-0.2440, 0.0458) |
| BMI |  |  | 0.0083, 0.2258  (-0.0055, 0.0221) |
| AICc, BIC | -57.16, -48.20 | -36.35, -21.56 | -27.24, -10.70 |

^a^first three-day sampling results used for the linear mixed model as repeated-measures data

^b^adjusting for age and race/ethnicity

^c^adjusting for age, race/ethnicity, and BMI

^d^information of age and race/ethnicity were obtained from our previous study (Chen et al. 2025b)

^e^information of BMI was obtained from our previous study (Chen et al. 2025a)

**p-value<0.01

*p-value<0.05

Table S4 Association between lifetime hand-arm vibration exposure dose (a_hv-lifetime_) and passive stiffness (PS) among the exposure group only after adjusting for none, demographic characteristics, or demographic and health characteristics in the linear mixed model.

|  |  | β, p-value  (95% CI) |  |
| --- | --- | --- | --- |
|  | Simple^a^ | Demographic^a, b, d^ | Demographic  +health status^a, c, d, e^ |
| Right Hand |  |  |  |
| 𝑎_ℎ𝑣−lifetime_ | 4.752, 0.2304  ( -3.350, 12.85) | 4.318, 0.3331  (-5.010, 13.65) | 1.683, 0.6479  (-6.207, 9.572) |
| Age |  | 0.8056, 0.6589  (-3.072, 4.683) | 2.158, 0.1830  (-1.184, 5.499) |
| Race/ethnicity-Afr |  | -46.04, 0.4156  (-165.0, 72.92) | -158.7, 0.0234*  (-291.6, -25.94) |
| Race/ethnicity-His |  | 1.948, 0.9708  (-111.5, 115.3) | -26.55, 0.5517  (-121.7, 68.62) |
| BMI |  |  | 12.59, 0.0195*  (2.443, 22.73) |
| AICc, BIC | 580.0, 586.7 | 564.6, 574.8 | 556.4, 567.4 |
| Left Hand |  |  |  |
| 𝑎_ℎ𝑣−lifetime_ | 5.874, 0.2030  (-3.532, 15.28) | 5.309, 0.2778  (-4.865, 15.48) | 4.966, 0.3255  (-5.653, 15.58) |
| Age |  | 1.251, 0.4384  (-2.149, 4.650) | 1.554, 0.3730  (-2.130, 5.238) |
| Race/ethnicity-Afr |  | -83.97, 0.0962  (-185.3, 17.38) | -111.5, 0.1142  (-254.4, 31.53) |
| Race/ethnicity-His |  | -29.76, 0.5256  (-128.9, 69.42) | -37.51, 0.4542  (-143.9, 68.90) |
| BMI |  |  | 3.027, 0.5454  (-7.652, 13.71) |
| AICc, BIC | 585.3, 592.0 | 567.6, 577.8 | 565.1, 576.1 |

^a^first three-day sampling results used for the linear mixed model as repeated-measures data

^b^adjusting for age and race/ethnicity

^c^adjusting for age, race/ethnicity, and BMI

^d^information of age and race/ethnicity were obtained from our previous study (Chen et al. 2025b)

^e^information of BMI was obtained from our previous study (Chen et al. 2025a)

*p-value<0.05

Table S5 Association between lifetime hand-arm vibration exposure dose (a_hv-lifetime_) and the all-six-day passive stiffness (PS) after adjusting for none, demographic characteristics, or demographic and health characteristics in the linear mixed model.

|  |  | β, p-value  (95% CI) |  |
| --- | --- | --- | --- |
|  | Simple^a^ | Demographic^a, b, d^ | Demographic  +health status^a, c, d, e^ |
| Right Hand |  |  |  |
| 𝑎_ℎ𝑣−lifetime_ | 7.253, 0.0045**  (2.472, 12.03) | 7.606, 0.0057**  (2.452, 12.76) | 6.801, 0.0087**  (1.913, 11.69) |
| Age |  | -0.1474, 0.8901  (-2.335, 2.040) | -0.0470, 0.9624  (-2.095, 2.001) |
| Race/ethnicity-Afr |  | -36.40, 0.3021  (-107.8, 35.04) | -81.35, 0.0482*  (-162.0, -0.6998) |
| Race/ethnicity-His |  | -18.82, 0.5693  (-86.37, 48.73) | -29.15553, 0.3545  (-93.19, 34.88) |
| BMI |  |  | 6.234, 0.0510  (-0.0297, 12.50) |
| AICc, BIC | 1775.9, 1788.0 | 1761.9, 1782.6 | 1756.0, 1779.5 |
| Left Hand |  |  |  |
| 𝑎_ℎ𝑣−lifetime_ | 7.923, 0.0128*  (1.840, 14.00) | 8.475, 0.0120*  (2.053, 14.90) | 8.206, 0.0137*  (1.861, 14.55) |
| Age |  | 0.2136, 0.8424  (-1.987, 2.414) | 0.2629, 0.8036  (-1.908, 2.434) |
| Race/ethnicity-Afr |  | -53.93, 0.1295  (-124.9, 17.08) | -84.35, 0.0507  (-169.0, 0.2812) |
| Race/ethnicity-His |  | -32.68, 0.3297  (-100.7, 35.32) | -40.34, 0.2316  (-108.4, 27.75) |
| BMI |  |  | 4.150, 0.1979  (-2.341, 10.64) |
| AICc, BIC | 1779.2, 1791.2 | 1763.3, 1784.0 | 1759.7, 1783.1 |

^a^all-six-day sampling results used for the linear mixed model as repeated-measures data

^b^adjusting for age and race/ethnicity

^c^adjusting for age, race/ethnicity, and BMI

^d^information of age and race/ethnicity were obtained from our previous study (Chen et al. 2025b)

^e^information of BMI was obtained from our previous study (Chen et al. 2025a)

**p-value<0.01

*p-value<0.05

Table S6 Association between lifetime hand-arm vibration exposure dose (a_hv-lifetime_) and the passive stiffness (PS) after adjusting for demographic and health characteristics, with or without adjusting for the interaction between a_hv-lifetime_ and BMI in the linear mixed model.

|  | β, p-value  (95% CI) | | |
| --- | --- | --- | --- |
|  | Demographic  +health status^a, c, d, e^ |  | Demographic  +health status  +BMI·a_hv-lifetime_^a, c, d, e^ |
| Right Hand |  |  |  |
| 𝑎_ℎ𝑣−lifetime_ | 6.972, 0.0128*  (1.643, 12.30) |  | 14.31, 0.4458  (-24.06, 52.67) |
| Age | 0.4434, 0.6840  (-1.790, 2.6771) |  | 0.3650, 0.7465  (-1.957, 2.687) |
| Race/ethnicity-Afr | -81.42, 0.0679  (-169.4, 6.531) |  | -82.44, 0.0710*  (-1.726, 7.751) |
| Race/ethnicity-His | -21.47, 0.5294  (-91.30, 48.35) |  | -29.16, 0.5464  (--92.56, 50.48) |
| BMI | 7.497, 0.0330*  (0.6671, 14.33) |  | 8.990, 0.0871  (-1.434, 19.41) |
| BMI·a_hv-lifetime_ |  |  | -0.2396, 0.6914  (-1.481, 1.001) |
| AICc, BIC | 889.0, 905.6 |  | 890.6, 908.8 |
| Left Hand |  |  |  |
| 𝑎_ℎ𝑣−lifetime_ | 9.039, 0.0108*  (2.317, 15.76) |  | 52.95, 0.0163*  (10.85, 95.04) |
| Age | 0.0724, 0.9484  (-2.227, 2.372) |  | -0.2764, 0.7910  (-2.423, 1.870) |
| Race/ethnicity-Afr | -94.37, 0.0401*  (-184.0, -4.702) |  | -96.64, 0.0243*  (-179.4, -13.91) |
| Race/ethnicity-His | -48.78, 0.1743  (-120.9, 23.36) |  | -41.95, 0.2054  (-108.8, 24.90) |
| BMI | 3.833, 0.2594  (-3.044, 10.71) |  | 10.76, 0.0232*  (1.629, 19.89) |
| BMI·a_hv-lifetime_ |  |  | -1.458, 0.0397*  (-2.841, -0.0754) |
| AICc, BIC | 901.5, 918.1 |  | 898.5, 916.7 |

^a^first-three-day sampling results used for the linear mixed model as repeated-measures data

^b^adjusting for age and race/ethnicity

^c^adjusting for age, race/ethnicity, and BMI

^d^information of age and race/ethnicity were obtained from our previous study (Chen et al. 2025b)

^e^information of BMI was obtained from our previous study (Chen et al. 2025a)

*p-value<0.05

| Table S7 Estimated Power for Left Hand Models. | | |
| --- | --- | --- |
| **Effect Size** | **Power** | **MCSE*** |
| 2.00 | 0.0909 | 0.0029 |
| 2.50 | 0.1136 | 0.0032 |
| 3.00 | 0.1547 | 0.0036 |
| 3.50 | 0.1876 | 0.0039 |
| 4.00 | 0.2358 | 0.0042 |
| 4.50 | 0.2767 | 0.0045 |
| 5.00 | 0.3190 | 0.0047 |
| 5.50 | 0.3980 | 0.0049 |
| 6.00 | 0.4511 | 0.0050 |
| 6.50 | 0.5085 | 0.0050 |
| 7.00 | 0.5685 | 0.0050 |
| 7.50 | 0.6259 | 0.0048 |
| 7.85 | 0.6649 | 0.0047 |
| 8.00 | 0.6800 | 0.0047 |
| 8.50 | 0.7388 | 0.0044 |
| 9.00 | 0.7773 | 0.0042 |
| 9.50 | 0.8215 | 0.0038 |
| 10.00 | 0.8534 | 0.0035 |
| *Monte Carlo Standard Error | | |


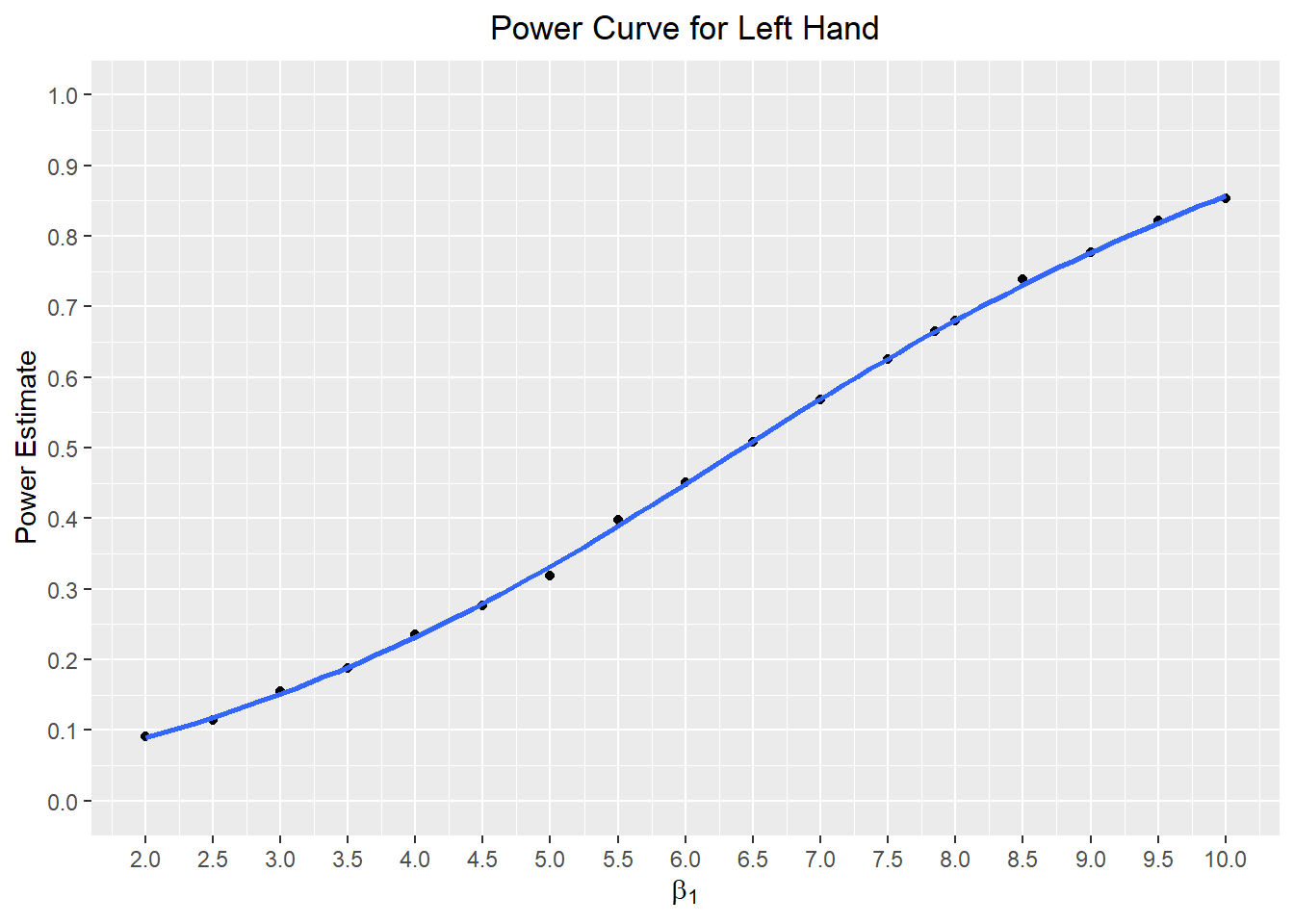


Figure S1. Estimated power curve for the left hand models.

| Table S8 Estimated Power for Right Hand Models. | | |
| --- | --- | --- |
| **Effect Size** | **Power** | **MCSE*** |
| 2.00 | 0.0971 | 0.0030 |
| 2.50 | 0.1139 | 0.0032 |
| 3.00 | 0.1548 | 0.0036 |
| 3.50 | 0.1893 | 0.0039 |
| 4.00 | 0.2309 | 0.0042 |
| 4.50 | 0.2692 | 0.0044 |
| 5.00 | 0.3284 | 0.0047 |
| 5.50 | 0.3944 | 0.0049 |
| 6.00 | 0.4546 | 0.0050 |
| 6.50 | 0.5144 | 0.0050 |
| 7.00 | 0.5691 | 0.0050 |
| 7.50 | 0.6330 | 0.0048 |
| 7.85 | 0.6735 | 0.0047 |
| 8.00 | 0.6745 | 0.0047 |
| 8.50 | 0.7438 | 0.0044 |
| 9.00 | 0.7895 | 0.0041 |
| 9.50 | 0.8258 | 0.0038 |
| 10.00 | 0.8664 | 0.0034 |
| *Monte Carlo Standard Error | | |


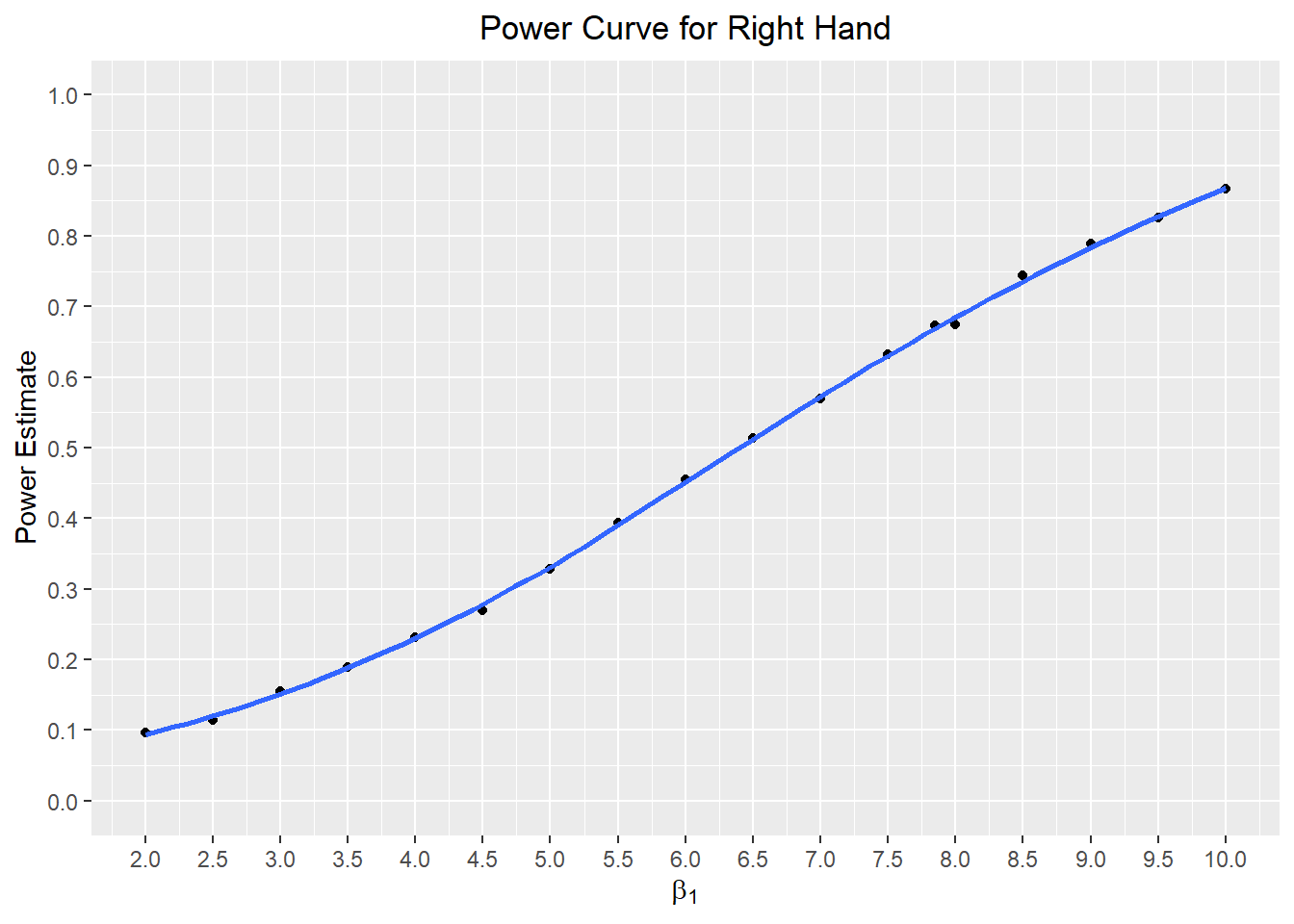


Figure S2. Estimated power curve for the right hand models.
